# Molecular Diet Analysis of Adélie Penguins (*Pygoscelis adeliae*) in the Ross Sea Using Fecal DNA

**DOI:** 10.3390/biology11020182

**Published:** 2022-01-24

**Authors:** Nazia Tabassum, Ji-Hyun Lee, Soo-Rin Lee, Jong-U Kim, Hyun Park, Hyun-Woo Kim, Jeong-Hoon Kim

**Affiliations:** 1 Industry 4.0 Convergence Bionics Engineering, Pukyong National University, Busan 48513, Korea; naziaat110@gmail.com (N.T.); srlee090@pukyong.ac.kr (S.-R.L.); 2 Department of Marine Biology, Pukyong National University, Busan 48516, Korea; jhlee208@pukyong.ac.kr; 3 Division of Life Science, Korea Polar Research Institute, Incheon 21990, Korea; wildlife@kopri.re.kr; 4 Department of Biotechnology, Korea University, Seoul 02841, Korea; hpark@korea.ac.kr; 5 Marine Integrated Biomedical Technology Center, National Key Research Institutes in Universities, Pukyong National University, Busan 48513, Korea

**Keywords:** *Pygoscelis adeliae*, Ross Sea, NGS, metabarcoding, diet analysis

## Abstract

**Simple Summary:**

The diet of Adélie penguins, *Pygoscelis adeliae*, in the Ross Sea was studied applying quantitative polymerase chain reaction (qPCR) protocols to their feces. Two krill species (*Euphausia superba* and *Euphausia crystallorophias*) and notothenioid fish (mainly *Pleuragramma antarctica* and *Pagothenia borchgrevinki*) were among the most abundant components of the diet. The composition of the two krill species and notothenioid fish was found to be strongly related to the geographic characteristics of the Ross Sea.

**Abstract:**

The diet of Adélie penguins, *Pygoscelis adeliae*, is a useful indicator in understanding the ecological conditions of their habitats. The diets of Adélie penguins were studied using metabarcoding and quantitative PCR (qPCR) analyses of fecal DNA from seven habitats along the Ross Sea region. Using metabarcoding analysis with dual universal primers (18Sv9 and miniFish), the overall diet composition and detailed information about piscine prey were clearly elucidated. It was found that two krill species (*Euphausia superba* and *Euphausia crystallorophias*) and notothenioid fish were the most abundant in the diets of Adélie penguins. Among the notothenioid prey, *Pleuragramma antarctica* (56.50%) and *Pagothenia borchgrevinki* (18.21%) were the two most abundant species. qPCR analysis showed a significant geographic difference in the composition of main prey. Penguins inhabiting outbound parts of the Ross Sea (Capes Adare (CA) and Duke of York Island (DY)) mainly preyed on *E. superba*, without any significant changes in prey composition. By contrast, those inhabiting the inbound parts of the Ross Sea (Edmonson Point (EP) and Inexpressible Island (II)) preyed on *E. crystallorophias* and notothenioid fish rather than *E. superba.* Compared with the outbound habitats, prey compositions for penguins inhabiting the inbound regions were significantly different year to year, which was presumably due to the food availability based on the annual environmental and meteorological conditions of the coastal region along with the inbound parts of the Ross Sea.

## 1. Introduction

The Adélie penguin, *Pygoscelis adeliae*, is known to be the most abundant and widely spread penguin species in Antarctica, distributed along the entire coast of the continent [1]. As an omnivorous predator, it is regarded as a vital biomarker for ecological and environmental factors [2]. Thus, understanding the penguin’s feeding ecology can provide basic information to establish models and plans for sustainability of the species in each habitat. Previous studies of the diets of Adélie penguins were conducted either directly via visual inspection [3,4,5,6] or indirectly via stable isotopes and fatty acid composition [7,8]. Based on the combination of direct and indirect methods, it is known that the main diet of Adélie penguins includes krill and notothenioid fish and glacial squid [9,10].

Traditionally, penguin diets have been identified by the morphological classification of ingesta collected by a stomach flushing technique according to standard methods of the CCAMLR Ecosystem Monitoring Program (CEMP) (CCAMLR, 2014). However, these traditional methods have several limitations for long-term or frequent surveys, mainly because of the high cost and labor, limited accessibility to target species, the occasional adverse environmental impact on the site during sample collection, or administrative approval [11,12]. It is extremely difficult to identify partially digested prey to the species level, which is one of the main limiting factors in morphological diet studies. As DNA sequencing technology advanced, the metabarcoding technique was developed, in which the whole taxon within the sample can be analyzed immediately with relatively low cost and short analytic time using the next-generation sequencing (NGS) platform. Compared with traditional visual analysis of prey remains, much higher numbers of diet samples can be rapidly and accurately processed at relatively low cost and with less labor using metabarcoding analysis [13]. There have been several metabarcoding studies on the diets of Adélie penguins [6,14,15]. However, some of them used the remaining contents by the stomach-flushing technique, which made it challenging to obtain sufficient sample numbers for analysis. In addition, this technique may have adverse impacts on both the examined individuals and their habitats, occasionally causing death by post-traumatic shock or asphyxia following stomach flushing [16]. Alternatively, metabarcoding analysis of fecal DNA can be applied when it is difficult to access a predator’s stomach contents [6,15]. However, such studies depend solely on a single marker, a short 18S region, which makes it challenging to know the exact species name or precise quantitative value of each prey item.

We investigated the diets of Adélie penguins from seven habitats along the Ross Sea, Antarctica, by metabarcoding analysis of isolated fecal DNA, which was collected for three years (2017 to 2019). Dual universal primers (18Sv9 for eukaryotes and mini fish for fish taxa) were used for the metabarcoding analysis to obtain more precise information of the diets. In addition, quantitative polymerase chain reaction (qPCR) was applied for higher accuracy in quantitative analysis of the main prey items of Adélie penguins: notothenioid fish, *Euphausia superba*, and *Euphausia crystallorophias*. The results show geographic variability in prey items, which is related to their availability and abundance. These results can help to expand our knowledge of the relationship between penguin populations and environmental conditions in the Ross Sea.

## 2. Materials and Methods

### 2.1. Sample Collection

**Figure 1:**
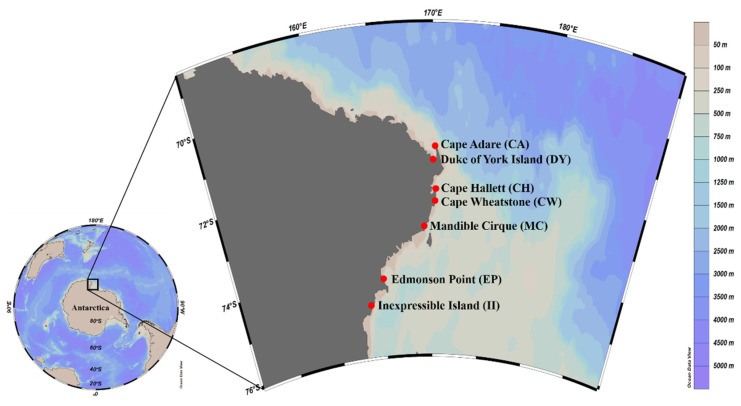
Sample sites for fecal DNA analysis in the Ross Sea region. Map view created by Ocean Data View software version 5.1.7 (Schlitzer, R., Ocean Data View, 2018, odv.awi.de, accessed on 23 December 2021).

Fresh scat samples from individual Adélie penguins were collected from their breeding colonies in the Ross Sea Region Marine Protected Area, including Cape Adare (CA), Duke of York Island (DY), Cape Hallett (CH), Cape Wheatstone (CW), Mandible Cirque (MC), Edmonson Point (EP), and Inexpressible Island (II) (Figure 1 and Table 1). The size of Adélie penguin colonies in the area where the samples were collected varied from 1890 pairs at Edmonson Point to 227,000 pairs at Cape Adare (Table 1) [17,18]. Sample collections were conducted during the guard stage [19] in 3 consecutive breeding seasons, from December 2017 to January 2020. However, scat samples were collected only at CH and II for all 3 years, whereas 2- or 1-year samples were collected at the rest of the sites (Table 1). Except for samples collected at 3 sites (CA, CH, and II) in January 2020, all other scat samples were collected in December. At each sampling site, 11 scat samples were collected, with several exceptions: 5 at II in 2017, 7 at CW in 2018, and 10 at CH in 2017 (Table 1). Only freshly excreted unfrozen scat was collected around the nests, minimizing the impact on the penguins’ incubation process and colonies.

### 2.2. Fecal DNA Isolation

Immediately after collection, the scat samples were put into 6 volumes of lysis buffer (50 mM Tris-HCl pH 8.0, 500 mM NaCl, and 50 mM EDTA) and stored frozen at −20 °C until delivery to the laboratory for further analysis. Fecal DNA was extracted using the AccuPrep genomic DNA extraction kit (Bioneer, Daejeon, Republic of Korea) following the manufacturer’s instructions. The frozen samples in lysis buffer were homogenized using a FastPrep-24™ Classic homogenizer (MP Biomedicals) after adding sodium dodecyl sulfate (2%). The quantity and integrity of extracted DNA were analyzed using a NanoDrop 1000 spectrophotometer (Thermo Fisher Scientific, Waltham, MA, USA). Extracted fecal DNA was aliquoted and stored at −70 °C until being used for library preparation and qPCR analysis.

### 2.3. Primer Design and PCR Amplification of Prey Items

Metabarcoding analyses were conducted with two universal primers to amplify trace amounts of diet from fecal DNA. A eukaryotic-specific universal primer set targeting the V9 region of 18S rRNA (18Sv9) was adopted for the overall prey composition, with an amplicon size of approximately 115–140 bp [20]. For the fish taxa, we initially tested the MiFish primer set, which has been widely used for environmental DNA (eDNA) metabarcoding analysis of fish taxa [21]. However, it showed a high degree of cross-reactivity to host DNA, presenting little prey DNA in the metabarcoding results (data not shown). Thus, another fish-specific primer set targeting the same region was designed by multiple alignment of the currently reported 12S region of 122 Antarctic fish and Adélie penguins using Geneious software [22]. The barcode size amplified by the primer set, named “miniFish”, was approximately 91 bp (Table 2).

### 2.4. Library Construction and NGS

Two PCR steps were used to construct the NGS library; 18Sv9 and miniFish primers were used for eukaryotic and fish taxa, respectively (Table 2). The first PCR for the MiSeq platform was conducted with primers overhanging the linker sequence for the Nextera XT Index Kit (Illumina, San Diego, CA, USA). The first PCR mixtures (20 μL) contained 1 μL of a template (5–20 ng/μL conc.), 1 μL F/R primer (10 pmol) each, 2 μL dNTPs (each 2.5 mM), 2 μL 10× Ex Taq Buffer, 0.2 μL ExTaq HS DNA polymerase (Takara, Shiga, Japan), and DNase/RNase free water. PCR amplification was performed under the following cycling conditions: initial denaturation at 94 °C for 3 min, followed by 20 cycles of 94 °C for 30 s, then 60 °C for 30 s for 18Sv9 primers or 55 °C for 30 s for miniFish, 72 °C for 30 s, with a final extension of 3 min at 72 °C. The amplified PCR products were stained with loading STAR (Dynebio, Republic of Korea) after 1% agarose gel electrophoresis. The amplicons of the expected size on the gel (approximately 230 bp for 18Sv9 and 200 bp for miniFish) were purified using an AccuPrep Gel Purification Kit (Bioneer, Daejeon, Republic of Korea) for the second PCR.

The second PCR amplification was conducted using a Nextera XT Index Kit (Illumina, San Diego, CA, USA) following the manufacturer’s instructions. The constructed libraries were identified by 1.5% agarose gel electrophoresis (approximately 270-300 bp) and further purified using the AccuPrep Gel Purification Kit (Bioneer, Daejeon, Republic of Korea). The integrity and quantity of the constructed libraries were measured using a Quantus fluorometer (Promega, Madison, WI, USA) and a BioAnalyzer 2100 (Agilent Technologies, Palo Alto, CA, USA). The indexed libraries were sequenced on the Illumina MiSeq platform using a MiSeq Reagent v3 600-cycle kit (Illumina, San Diego, CA, USA).

### 2.5. Bioinformatics Analysis

After adapter/index sequences were trimmed from the raw MiSeq reads and reads with a QV below 20, fewer than 100 nucleotides were discarded using CLC Genomics Workbench v.8.0 (CLC Bio, Cambridge, MA, USA). The trimmed raw reads were further merged using mothur (v.1.43.0) software [23]. The merged reads were screened with criteria of 7 bp overlap, zero mismatches, trimmed primer sequences, and expected size (115–140 bp for eukaryotes and 91 bp for fish taxa). The merged reads were clustered into operational taxonomic units (OTUs) at 99.6% of sequence identity. OTU clustering and the removal of chimeric sequences were conducted using UCHIME [24]. The OTUs were annotated with BLASTN (v.2.7.1) against the NCBI NT sequence database. Species names were assigned for each OTU with a sequence identity higher than or equal to 99%. Those whose identity ranged from 90 to 99% in the 18Sv9 metabarcoding and from 95 to 99% in the miniFish metabarcoding received genus names. OTUs with lower identities were considered “unknown”. Nonmetric multidimensional scaling (NMDS) analysis was performed based on the Bray–Curtis distance of phylum and genus proportions from the 18Sv9 and miniFish metabarcoding, respectively.

### 2.6. qPCR Analysis

qPCR was performed using 3 designed specific primers to estimate the abundance of fish taxa and 2 krill species (*E. superba* and *E. crystallorophias*) in the fecal DNA sample sets (Table 2). A standard curve was constructed for each primer set using the serially diluted plasmid harboring each target sequence, as described in a previous study [25]. qPCR was conducted using a Mic qPCR cycler system (BioMolecular Systems, Upper Coomera, Australia). The qPCR mixture in a 20 μL volume included 2 μL genomic DNA, 1 μL each of forward and reverse primer (10 pmol), 6 μL DNase/RNase free water, and 10 μL 2× Luna Universal qPCR master mix (New England Biolabs, USA). PCR conditions for fish taxa included initial denaturation at 95 °C for 5 min, followed by 40 cycles at 95 °C for 5 s, 55 °C, for 20 s, and 72 °C for 20 s, and a final extension at 72 °C for 3 min, while those for the 2 krill consisted of initial denaturation at 94 °C for 5 min, followed by 40 cycles at 94 °C for 30 s, 64.9 °C for 15 s, and 72 °C for 15 s, and a final extension at 72 °C for 5 min. A melting curve was generated after the final extension step of each assay in a 65 to 95 °C melting gradient at a rate of 0.3 °C/s. The fidelity of each qPCR assay was verified with corresponding fish and krill genomic DNA as positive control and water as negative control. Additionally, the specificity of the 2 krill-specific primer sets was confirmed by negative amplification for nontarget krill species, whereas the specificity of the miniFish primers was established using metabarcoding analysis. The calculation of copy numbers was based on Ct values of samples in each standard curve. Each copy number was normalized by dividing the 18S rRNA gene copy number as an endogenous control.

## 3. Results

### 3.1. Fecal DNA Metabarcoding of Pygoscelis Adeliae in the Ross Sea Region Using 18Sv9 Universal Primers

**Figure 2:**
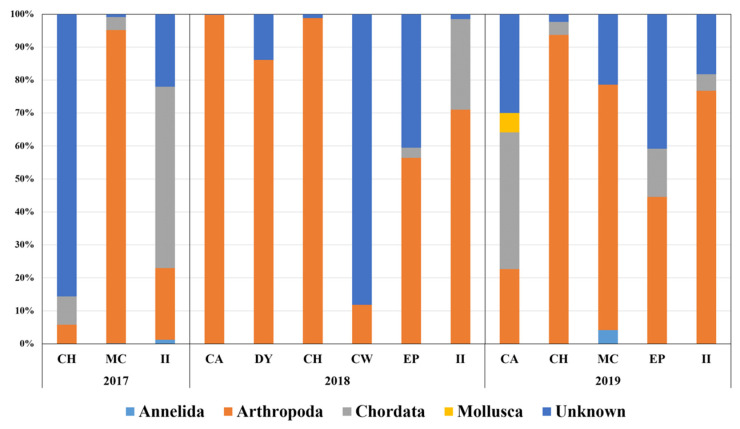
Metabarcoding of fecal DNA using 18Sv9 universal primer set. Graph displays proportions of phyla of prey items at different sites by year.

In total, 143 fecal DNA from seven sample sites were prepared during the surveys from December 2017 to January 2020. Metabarcoding analysis was conducted with 14 pooled samples from different habitats and years (Table 1). To know the overall diet composition of Adélie penguins, the first metabarcoding analysis was performed using the 18Sv9 universal primer set. On average, 204,657 raw reads were generated from each pooled sample based on MiSeq sequencing. The lowest and highest raw read numbers were obtained at II in 2018 (65,911) and MC in 2017 (413,458) (Table 3). After trimming, more than 80% of the raw reads for each site were used for further analysis as the reliable “merged reads,” supporting the quality of NGS reads (Table 3). Despite their specificity for eukaryotic taxa, the merged reads generated by the 18Sv9 universal primer set still presented a high number of bacterial taxa, whose proportion in pooled samples ranged from 24.72 to 68.17%. In addition to bacterial sequences, the ratio of penguin DNA was high, ranging from 17.54 to 50.07%. After eliminating the bacterial and penguin DNA, only tiny proportions (0.07–31.15%) were obtained as putative prey items of Adélie penguins (Table 3). We failed to identify the species level, mainly because of low sequence variability in the 18Sv9 marker. Hence, prey items in each pooled sample were further classified into five phyla: Annelida, Arthropoda, Chordata, Mollusca, and Unknown (Figure 2). Among the five phyla, arthropods were most abundant, making up 61.33% on average, followed by unknown (26.20%) and chordates (11.66%). Two phyla, Annelida (0.39%) and Mollusca (0.43%), were negligible (Figure 2).

The arthropod OTUs belonged to either *E. superba* or *E. crystallorophias*, the average proportion of which among arthropod OTUs was 75.64%. *E. crystallorophias* (49.88%) was approximately two-fold higher than *E*. *superba* (25.14%; Figure 2). The copepod OTUs placed second, with an average proportion in arthropod OTUs of 16.92%. Among seven copepod genera, *Calanus* (7.78%) was the most abundant, followed by *Metridia* (4.60%) and *Paraeuchaeta* (1.90%) (Figure 2). All OTUs in the phylum Chordata showed the highest identity to notothenioid fish, which is the most abundant fish taxon in the Southern Sea. Only two OTUs in Annelida have identified either *Lumbrineris* sp. or *Tomopteris* sp., whose average proportion was only 0.40% among the five taxa. One OTU in Mollusca showed the highest identity to *Limacina helicina*, a tiny swimming planktonic sea snail, which was identified only one time at CA in 2019. Finally, an unknown OTU showed the highest identity to *Eimeria furonis*, suggesting putative gastrointestinal helminths. Although *Parorchites zederi* and the nematode *Stegophorus macronectes* are among the parasites commonly found in penguins in Antarctica [26], we failed to identify the exact species, mainly because of the lack of reference sequences in the database.

**Figure 3:**
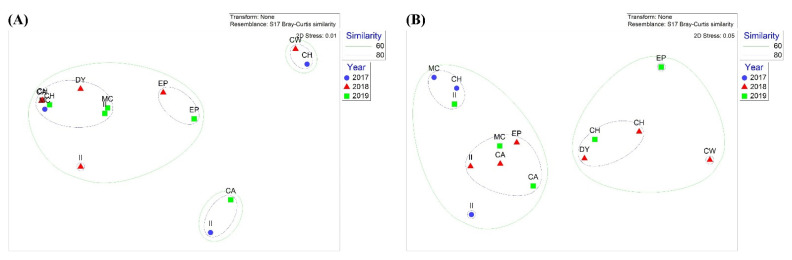
Nonmetric multidimensional scaling (NMDS) plot of (**A**) eukaryotic communities using 18Sv9 metabarcoding and (**B**) fish communities using miniFish metabarcoding in different years. Solid lines indicate 60% and 80% similarity.

Nonmetric multidimensional scaling (NMDS) analysis was conducted to compare community structures obtained by metabarcoding (Figure 3). Three clusters were observed via NMDS in the 18Sv9 metabarcoding analysis (Figure 3A). The first group (CW in 2018 and CH in 2017) was characterized by a high number of unknown parasites, mainly for those starved. The second group (CA in 2019 and II in 2017) showed a high number of Chordata taxa, mostly fish, whereas the others (group 3) exhibited an arthropod-dominant group, most of them belonging to krill. These data support the suggestion that krill are major prey of Adélie penguins in the Ross Sea, with a few exceptional cases.

### 3.2. Fecal DNA Metabarcoding of Pygoscelis Adeliae in the Ross Sea Region Using miniFish Primers

**Figure 4:**
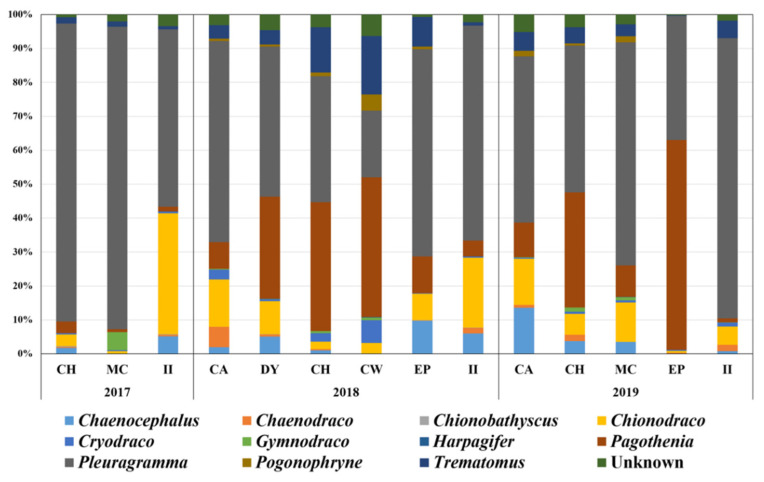
Prey items identified using miniFish metabarcoding. Graph displays proportions of prey items at the genus level at different sites by year.

Piscine prey items of *P*. *adeliae* were analyzed using the miniFish primer set (Figure 4). Approximately 1.3 million to 6.1 million raw reads were generated at each site. After trimming, the average proportion of qualified merged reads was 45.93%, ranging from 3.22 to 76.97% (Table 4). Interestingly, extremely low numbers of qualified merged reads were obtained at CA in 2018 (3.22%) and 2019 (8.11%). The proportion of non-fish reads ranged from 4.10 to 15.73%, indicating high specificity for piscine taxa.

Five families (Nototheniidae, Channichthyidae, Artedidraconidae, Bathydraconidae, and Harpagiferidae) covering 11 genera (*Chaenocephalus*, *Chaenodraco*, *Chionobathyscus*, *Chionodraco*, *Cryodraco*, *Gymnodraco*, *Harpagifer*, *Pagothenia*, *Pleuragramma, Pogonophryne*, and *Trematomus*) were detected as piscine prey of Adélie penguins via miniFish metabarcoding (Table 5). Of 22 haplotypes, 12 showed 100% sequence identity, indicating a high number of reference sequences for fish species in the Ross Sea. However, species names were unable to be assigned to three haplotypes from *Trematomus*, *Chinodraco*, and *Pogonophryne* because of the low variability to distinguish the relative species in each genus (Table 5). Among the 11 genera, the Antarctic silverfish, *P*. *antarctica,* was the most abundant, accounting for 56.50% of total piscine reads, followed by *Pagothenia borchgrevinki* (18.20%), *Chionodraco* spp. (9.61%), and *Trematomus* spp. (5.15%).

The NMDS result showed two clusters but failed to identify any patterns by years and habitats (Figure 3B). The NMDS analysis showed that two clusters were determined by the proportions of the two main fish species. The first cluster included the nine sites where *P*. *antarctica* was the most abundant. At the same time, *P*. *borchgrevinki* was equivalent to or higher than *P*. *antarctica* at the other five sites (CH, DY, and CW in 2018 and CH and EP in 2019), indicating no spatiotemporal pattern (Figure 3B).

### 3.3. qPCR Analysis of Main Prey Items

**Figure 5:**
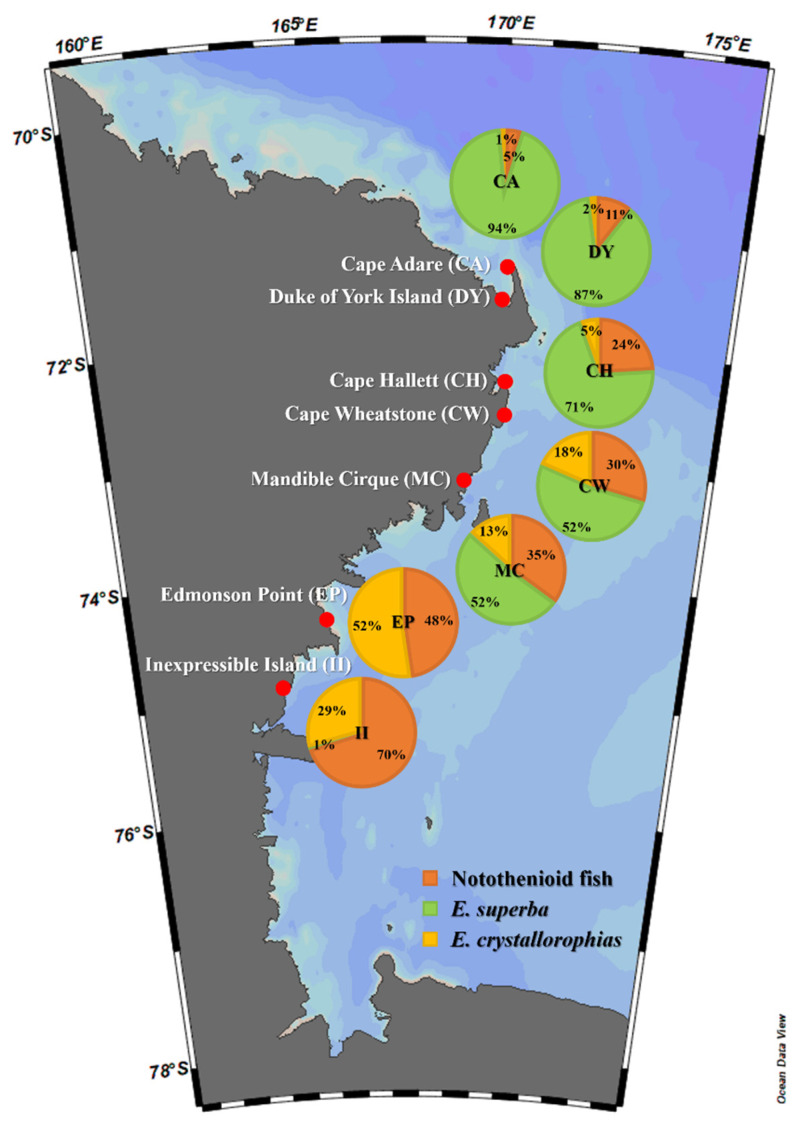
Geographic proportions of notothenioid fish, *Euphausia superba*, and *Euphausia crystallorophias* in fecal DNA of Adélie penguins (*Pygoscelis adeliae*) in the Ross Sea region.

On the basis of the metabarcoding analysis of fecal DNA using two primer sets, two krill species (*E*. *superba* and *E*. *crystallorophias*) and notothenioid fish, especially *P*. *antarctica* and *P*. *borchgrevinki*, were identified among the most abundant prey items of Adélie penguins (Figure 4). To conduct a more accurate quantitative analysis of those main prey items, qPCR was adopted for individual samples of fecal DNA. Three primer sets, including two krill-specific primer sets (*E*. *superba* and *E*. *crystallorophias*) and one fish-specific (miniFish), were used for the quantitative analysis. The proportions of the main prey items showed clear geographic patterns. Higher proportions of *E*. *superba* and *E*. *crystallorophias* were respectively identified in the outbound and inbound habitats in the Ross Sea (Figure 5 and Table 6). In fact, the proportions of *E*. *crystallorophias* showed a clear opposite pattern to those of *E*. *superba*, whose highest proportions were identified in the most inbound habitats, EP and II. The proportion of *E*. *superba* at those two habitats was less than 1% (Figure 5). By contrast, the proportion of *E*. *superba* was highest in the most outbound habitats, including CA and DY. The proportion of piscine diets showed a similar pattern with *E*. *crystallorophias*, in which the two most inbound habitats, EP and II, showed their highest proportions, while those in CA and DY were lower than 10% (Figure 5).

**Figure 6:**
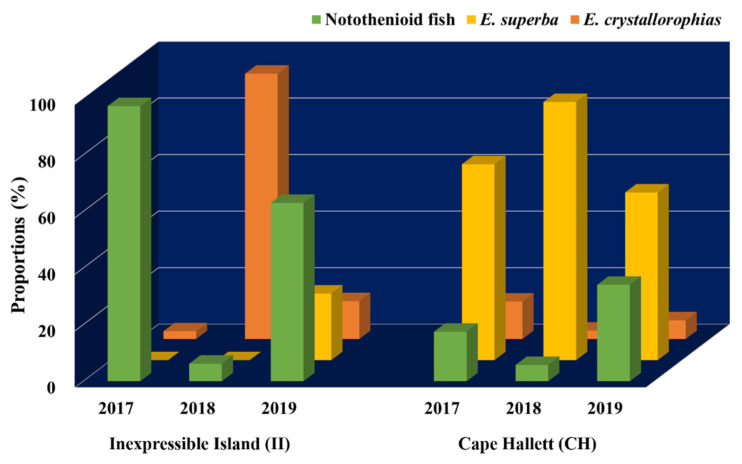
Proportions of main prey items (Notothenioid fish, *Euphausia superba*, and *Euphausia crystallorophisa*) in fecal DNA of Adélie penguins (*Pygoscelis adeliae*) at Inexpressible Island (II) and Cape Hallett (CH) for three successive years.

Annual proportional changes of the main prey items (Notothenioid fish, *E. superba*, and *E. crystallorophias*) were also analyzed at two sites (CH and II), where all 3-year scat samples were collected in the Ross Sea (Figure 6). Proportions of the main prey were similar for 3 years at the outbound habitat (CA), among which *E*. *superba* was the largest, followed by notothenioid fish and *E. crystallorophias*. By contrast, their proportions were highly variable year to year in the inbound habitat (II). In 2017 and 2019, a higher proportion of fish prey was identified, while a predominant proportion of *E. crystallorophias* was found in 2018 (Figure 6). Even between 2017 and 2019, we were able to identify differences in krill composition. Compared to 2017, much higher proportions of *E. superba* and *E. crystallorophias* were found in 2019. However, *E. crystallorophias* was the main krill prey of Adélie penguins in the inbound habitat, II (Figure 6).

## 4. Discussion

We analyzed the diets of Adélie penguins in the Ross Sea region by metabarcoding of fecal DNA with multiple markers (18Sv9 and miniFish). The barcode sizes amplified by the two primer sets were approximately 91–140 bp, which were much smaller than regular barcodes. The quality and quantity of dietary DNA remaining in the scat are often much lower compared to other environmental samples because of the vigorous digestion process [27]. Therefore, amplifying of short sequences, named “minibarcodes”, is used for samples containing degraded DNA such as processed food, water, soil, or scat [28,29]. Although it is one of the most widely used methods [30,31,32], mitochondrial COI minibarcodes show a high degree of cross-reactivity with bacterial DNA presenting most of their sequences, which outnumbered the trace amounts of degraded dietary DNA. We alternatively applied the nuclear 18Sv9 universal primer set, which was explicitly designed for eukaryotic taxa [33,34]. However, it also amplified a large amount of predator and bacterial DNA, accounting for up to 80% of total metabarcode reads (Table 3). Blocking primers are often used to suppress the host DNA in metabarcoding results [35,36]. We also tried them, but this attempt failed because they suppressed the DNA of penguins and dietary items (data not shown). Despite the short amplicon size and low sequence variability, metabarcoding analysis using the 18Sv9 primer set successfully showed the overall prey items of Adélie penguins in the Ross Sea at the phylum level, in which krill and notothenioid fish were the main prey items (Figure 2).

In addition to the diet study, 18Sv9 rRNA metabarcoding results of the penguins’ fecal DNA provided additional information. For instance, diverse putative parasites were identified, including Platyhelminthes (*Diphyllobothrium pacificum*, *Parorchites zederi*, *Parorchites* sp., *Spirometra erinaceieuropaei*, and *Zygobothrium* sp.), Chaetognatha (*Pseudosagitta* sp.), Tardigrada (*Acutuncus antarcticus*), Nematoda (*Contracaecum osculatum*), and Apicomplexa (*Eimeria* sp.). Many of them are among the previously reported parasites of Adélie penguins. *P. zederi* is one of the common parasites in Adélie penguins [26], and *D. pacificum* is one of the most widely identified parasites among the animals inhabiting Antarctica [37]. *Eimeria* sp. is also one of the three main Coccidian parasites (Apicomplexa) of penguins inhabiting Livingston Island and King George Island [38]. It is generally known that parasite composition is highly related to diet composition [39,40], and in particular, euphausiid species are known to be intermediate hosts for parasite species of Adélie penguins [26]. Further studies should be conducted to determine the relationship between diet composition and parasitic species, which may provide helpful information about their ecological implications in the Ross Sea. Moreover, parasite composition should be further monitored to estimate the health condition of Adélie penguins. Although *Dibothriocephalus*, *Adenocephalus*, and *Diphyllobothrium* are considered commensal parasites, the larva of some *Spirometra* can cause life-threatening diseases to host species [41]. Therefore, monitoring them with the use of molecular tools would be important not only in terms of their ecological implications but also for the sustainability of penguin colonies.

While the 18Sv9 primer set presented the overall prey items of Adélie penguins in the Ross Sea at the phylum level, its low sequence variability and short barcode size made it possible to analyze up to the species level. We obtained detailed information regarding the penguins’ fish diet using a fish-specific universal primer, miniFish, targeting their mitochondrial 12S region. The newly designed miniFish primer set showed a high degree of specificity for fish taxa (Notothenioidei) in the Southern Sea without amplifying either penguin or bacterial DNA (Table 4). Its amplicon size was also short (91 bp), which is suitable for amplifying fish sequences from highly degraded DNA samples isolated from scat, soil, or water. The amplified region lies within the MiFish marker, which is applicable to the established reference database [21]. Most of the haplotypes obtained by miniFish showed a high degree of sequence identity, assigning each species name and supporting a high number of reference sequences for fish species inhabiting the Ross Sea. However, because of its short amplicon size, some species in three genera, *Chionodraco*, *Pogonophryne*, and *Trematomus*, were indiscriminate using miniFish metabarcoding (Table 5). Those weaknesses can be compensated by comparative analysis of regional fish assemblage data obtained via eDNA metabarcoding using a more extended barcode primer set such as MiFish [21]. The newly designed miniFish primer would be a valuable tool for diet studies of piscivorous predators, such as penguins and seals in Antarctica.

miniFish metabarcoding showed that *P*. *antarctica* was the most abundant fish prey of Adélie penguins, making up 56.50% on average, followed by *P*. *borchgrevinki* (18.21%), *Chionodraco* spp. (9.61%), and *Trematomus* spp. (5.15%), which is consistent with previous studies [42,43]. Since Adélie penguins are omnivorous predators, this result is reasonable, knowing that the Antarctic silverfish, *P*. *antarctica*, is the most abundant pelagic fish inhabiting the Ross Sea [44,45]. However, there are different reports regarding Adélie penguin diets in previous studies. Krill and *P*. *borchgrevinki* were found to be the main diet according to underwater camera surveillance [46]. Another study, based on isotope 13C and 15N values, revealed that euphausiids are the main food source [47]. Those results may reflect regional characteristics, and metabarcoding analysis covered all cases. We identified a higher proportion of *P*. *borchgrevinki* than *P*. *antarctica* at several sites (Figure 4). In addition, compared with krill, an equal or higher number of fish taxa were shown at II in 2017 and 2018 (Figure 2). These proportions changed from year to year, and long-term metabarcoding analysis using a dual primer set would be one efficient strategy to determine the relationship between the proportions of prey and the environmental characteristics in the Ross Sea. Although *P*. *antarctica* was identified as the main fish prey of Adélie penguins, it is worth noting that 11 fish genera have been identified as prey items for these penguins in the Ross Sea, some of which are actually too large to be their prey (Figure 4). Furthermore, two genera, *Chionodraco* and *Trematomus*, made up approximately 15% of fish prey on average. Different from the two small pelagic fish prey, these two are known as demersal fish species. Terra Nova Bay, a highly productive area in the Ross Sea, is an important spawning and nursery ground for several fish species [48]. Thus, it would also be worth investigating the relationship between the reproduction of notothenioids and the dynamics of Adélie penguins.

While Adélie penguins’ main diet was found to be krill and notothenioid fish, which is consistent with previous studies [6,15], neither hydrozoans nor cephalopods were identified from fecal DNA in this study (Figure 2 and Table 3). Recent studies showed that hydrozoans have increasingly grown as penguin prey, which is presumably because of climate change [6,15]. Cephalopod *Psychroteuthis glacialis* was also considered to be among the prey items of Adélie penguins [49]. However, these differences may come from the seasonal availability of non-fish taxa in some habitats, especially those located inbound of the Ross Sea. In previous studies, except for fish taxa, a high degree of seasonal changes in the penguins’ diet was shown, which was presumably due to food availability by periodic changes in ice-covered regions along the inner coastal part of the Ross Sea such as polynyas [49,50]. Subsequently, more frequent and long-term surveys of Adélie penguins in the Ross Sea are required in order to understand the relationship between environmental changes and food availability in the region, and a long-term molecular survey of scat samples would be one of the most cost-effective strategies.

According to previous research, krill was found to be the most important prey of Adélie penguins in the Ross Sea. One of the most interesting findings by molecular analysis was a clear geographic difference of krill prey within the Ross Sea [51,52]. Higher proportions of *E*. *superba* were identified in the more outbound habitats, while the proportion of *E*. *crystallorophias* increased in the more inbound habitats (Figure 5). Differences in the abundance of two krill, *E. superba* and *E. crystallorophias*, are due to their availability in the Ross Sea. *E*. *superba* is considered a pelagic species in offshore areas, whereas *E*. *crystallorophias* is limited to inshore waters under ice [53]. Therefore, outbound habitats such as CA and DY would be favorable for *E*. *superba*, while the coastal waters near two inbound habitats, EP and II, may provide favorable conditions for *E*. *crystallorophias* rather than *E*. *superba.* We also identified that the prey of Adélie penguins inhabiting outbound habitats showed a rather stable pattern; the main prey was *E. superba*, with small amounts of fish species (Figure 6). By contrast, those in inbound habitats of the Ross Sea face a high degree of variation in their prey according to environmental changes. One of the most important environmental changes is the dynamics of the ice pack along the Ross Sea. Since they prefer being underneath the ice pack, changes in the availability of *E*. *crystallorophias* or fish may be related to polynya. Previous studies showed that the population of Adélie penguins is directly linked to sea ice [54] and temperature fluctuations [55]. This ecological indicator’s productivity has been linked to Antarctic krill [56], which is also sea ice-bound [57,58]. This could help researchers better understand the population dynamics of Adélie penguins, especially among the inbound habitats in the Ross Sea. Collectively, we established a pipeline to analyze the diets of Adélie penguins from scat samples in the Ross Sea using molecular techniques. We were able to obtain detailed information on the prey species of Adélie penguins. We also found that the penguins’ diets were highly affected by the geographic characteristics of the Ross Sea. Frequent and long-term surveys using this methodology would provide detailed information regarding the feeding ecology of Adélie penguins inhabiting the Ross Sea Region Marine Protected Area, which would be of practical use for their scientific conservation and management.

## 5. Conclusions

The diets of Adélie penguins (*P. adeliae*) were analyzed using fecal DNA with NGS and qPCR techniques. The combination of molecular tools used in this study successfully elucidated that two krill species and notothenioid fish are the penguins’ main diet species. However, diet compositions were highly variable according to the environmental conditions and geographic food availability. These data will help researchers expand our knowledge of the role of Adélie penguins in the ecosystem of the Ross Sea. Further studies should be conducted to understand the relationship between meteorological phenomena such as the occurrence of polynya and the diets of Adélie penguins in the Ross Sea.

## Figures and Tables

**Table 1:** Details of collection of Adélie penguin (*Pygoscelis adeliae*) fecal samples used in this study.

Site	GPS	Colony Size	2017	2018	2019	Total
Cape Adare (CA)	71.3355° S, 170.1397° E	227,000 [17]	0	11	11 *	22
Duke of York Island (DY)	71.6197° S, 170.0600° E	16,340 [18]	0	11	0	11
Cape Hallett (CH)	72.3166° S, 170.2166° E	42,628 [17]	10	11	11 *	32
Cape Wheatstone (CW)	72.5991° S, 170.2527° E	2746 [17]	0	7	0	7
Mandible Cirque (MC)	73.1631° S, 169.1647° E	16,837 [17]	11	0	11	22
Edmonson Point (EP)	74.3308° S, 165.1172° E	1890 [17]	0	11	11	22
Inexpressible Island (II)	74.9000° S, 163.6500° E	24,450 [17]	5	11	11 *	27
Total			26	62	55	143

* Samples collected in January 2020.

**Table 2:** Primers used in qPCR and NGS analysis of fecal DNA of Adélie penguins (*Pygoscelis adeliae*).

Name	Sequence (5′ to 3′)	Size (bp)	Description
Eus-F	CCCTTCCTTAACTCTCTTATTAGGAAGA	154	qPCR for *E. superba* (This study)
Eus-R	TGAAGAAGCACCGGCAATATGAAGC		
Euc-F	GAAGTCTAATTGGGGACGACCAG	207	qPCR for *E. crystallorophias* (This study)
Euc-R	CTAGTAAAAGAGTTAAGGAAGGAGGC		
miniFish-F	GTTATACGAGAGGCCCAAGTTG	133	qPCR for fish taxa (This study)
miniFish-R	TAAAGCCACTTTCGTGGTTG		
NGSmFish-F	TCGTCGGCAGCGTCAGATGTGTATAAGAGACAGGTTATACGAGAGGCCCAAGTTG	200	NGS for fish taxa (This study)
NGSmFish-R	GTCTCGTGGGCTCGGAGATGTGTATAAGAGACAGTAAAGCCACTTTCGTGGTTG		
NGS18Sv9-F	TCGTCGGCAGCGTCAGATGTGTATAAGAGACAGTTGTACACACCGCCCGTCGC	230	NGS for eukaryotes [20]
NGS18Sv9-R	GTCTCGTGGGCTCGGAGATGTGTATAAGAGACAGCCTTCYGCAGGTTCACCTAC		

**Table 3:** Reads of fecal DNAs generated using 18Sv9 universal primer set.

Year	Site	Raw Reads	Merged	Trimmed (%)	Total OTUs	Bacterial OTUs (%)	Penguin OTUs (%)	Other OTUs (%)	Unknown OTUs (%)	Prey OTUs (%)
2017	CH	206,799	191,923	172,527 (83.42)	1908	906 (47.48)	761 (39.88)	25 (1.31)	212 (11.11)	4 (0.20)
	MC	413,457	366,886	344,960 (83.43)	2521	819 (32.48)	771 (30.58)	316 (12.53)	172 (6.82)	443 (17.57)
	II	287,458	262,918	238,667 (83.02)	2863	1472 (51.41)	640 (22.35)	74 (2.58)	388 (13.55)	289 (10.09)
2018	CA	99,042	95,425	87,731 (88.57)	1351	334 (24.72)	567 (41.96)	26 (1.92)	57 (4.21)	367 (27.16)
	DY	136,739	131,310	122,607 (89.66)	2234	1523 (68.17)	392 (17.54)	25 (1.11)	289 (12.93)	5 (0.22)
	CH	112,246	108,092	100,432 (89.47)	1544	440 (28.49)	511 (33.09)	23 (1.48)	89 (5.76)	481 (31.15)
	CW	88,125	84,967	78,241 (88.78)	1252	486 (38.81)	652 (52.07)	20 (1.59)	93 (7.42)	1 (0.07)
	EP	80,022	77,651	71,633 (89.51)	1283	670 (52.22)	367 (28.60)	36 (2.80)	159 (12.39)	51 (3.97)
	II	65,911	64,148	58,519 (88.78)	1160	733 (63.18)	327 (28.18)	9 (0.77)	50 (4.31)	41 (3.53)
2019	CA	329,166	295,489	263,738 (80.12)	3647	1732 (47.49)	958 (26.26)	21 (0.57)	881 (24.15)	55 (1.50)
	CH	221,449	206,082	186,421 (84.18)	2063	647 (31.36)	498 (24.13)	22 (1.06)	829 (40.18)	67 (3.24)
	MC	217,619	202,090	177,117 (81.38)	1994	566 (28.38)	564 (28.28)	144 (7.22)	484 (24.27)	236 (11.83)
	EP	256,131	236,177	212,579 (82.99)	2666	1513 (56.75)	615 (23.06)	233 (8.73)	261 (9.78)	44 (1.65)
	II	351,038	319,669	294,189 (83.80)	3743	2323 (62.04)	771 (20.59)	38 (1.01)	564 (15.06)	48 (1.28)

**Table 4:** Metabarcoding reads of fecal DNAs generated using miniFish primer set.

Year	Site	Raw Reads	Merged	Trimmed (%)	Total OTUs	Nonfish OTUs (%)	Fish OTUs (%)
2017	CH	466,869	417,192	89,933 (19.26)	1539	90 (5.84)	1449 (94.15)
	MC	430,858	390,434	295,728 (68.63)	3223	308 (9.55)	2915 (90.44)
	II	411,949	375,381	299,065 (72.59)	6793	1123 (16.53)	5670 (83.46)
2018	CA	139,704	131,090	4,507 (3.22)	328	33 (10.06)	295 (89.93)
	DY	404,610	366,116	145,151 (35.87)	2759	301 (10.90)	2458 (89.09)
	CH	486,449	432,846	67,403 (13.85)	1780	213 (11.96)	1567 (88.03)
	CW	453,872	409,481	274,322 (60.44)	4939	777 (15.73)	4162 (84.26)
	EP	383,592	350,968	258,227 (67.31)	5075	417 (8.21)	4658 (91.78)
	II	613,755	540,098	397,584 (64.77)	7025	884 (12.58)	6141 (87.41)
2019	CA	366,387	332,391	29,732 (8.11)	1136	113 (9.94)	1023 (90.05)
	CH	546,858	484,040	203,386 (37.19)	3943	427 (10.82)	3516 (89.17)
	MC	535,567	475,415	261,071 (48.74)	5089	621 (12.20)	4468 (87.79)
	EP	355,283	327,315	273,474 (76.97)	2438	100 (4.10)	2338 (95.89)
	II	418,457	381,563	276,689 (66.12)	4121	450 (10.91)	3671 (89.08)

**Table 5:** Fish species determined using miniFish metabarcoding in Ross Sea.

Family	Species	Accession No.	Identity (%)	Query Cover
Nototheniidae	Trematomus loennbergii/lepidorhinus *	NC048965/MN864240	100/100	100
	*Pagothenia borchgrevinki*	KU951144, KX025131	100	100
	*Pleuragramma antarctica*	JF933905	100	100
	*Trematomus bernacchii*	MN841276	100	100
	*Trematomus pennellii*	MK007073	100	100
Channichthyidae	Chionodraco hamatus/rastrospinosus *	KT921282/ NC039543	100/100	100
	*Chaenocephalus aceratus*	JF933907	100	100
	*Chaenodraco wilsoni*	NC039158	100	100
	*Chionodraco myersi*	DQ526430	100	100
	*Cryodraco antarcticus*	NC045285	100	100
Artedidraconidae	Pogonophryne albipinna/scotti *	NC046024/LC069700	100/100	100
Bathydraconidae	*Gymnodraco acuticeps*	U90413	100	100

* Haplotypes with multiple species identities.

**Table 6:** Proportions of main prey items by quantitative analysis.

	CA	DY	CH	CW	MC	EP	II
Notothenioid fish (%)	4.84	10.57	24.09	29.57	35.04	47.68	70.11
*E*. *superba* (%)	93.76	87.40	70.54	52.00	51.66	0.00	0.45
*E*. *crystallorophias* (%)	1.40	2.03	5.37	18.43	13.30	52.31	29.44

## Data Availability

The sequencing data in this study are registered with the NCBI database under BioProject ID PRJNA749147.

## Abbreviations

qPCR	quantitative polymerase chain reaction
OTUs	operational taxonomic units
CA	Cape Adare
DY	Duke of York Island
CH	Cape Hallett
CW	Cape Wheatstone
MC	Mandible Cirque
EP	Edmonson Point
II	Inexpressible Island
